# The impact of COVID-19 on managing ophthalmic diseases: an international, descriptive study

**DOI:** 10.1007/s11845-023-03306-9

**Published:** 2023-02-11

**Authors:** Ethan Waisberg, Joshua Ong, Nasif Zaman, Sharif Amit Kamran, Alireza Tavakkoli, Andrew G. Lee

**Affiliations:** 1 University College Dublin School of Medicine, Belfield, Dublin 4 Ireland; 2 Michigan Medicine, University of Michigan, Ann Arbor, MI USA; 3 Human-Machine Perception Laboratory, Department of Computer Science and Engineering, University of Nevada, Reno, NV USA; 4 Department of Ophthalmology, Blanton Eye Institute, Houston Methodist Hospital, Houston, TX USA; 5 Center for Space Medicine, Baylor College of Medicine, Houston, TX USA; 6 The Houston Methodist Research Institute, Houston Methodist Hospital, Houston, TX USA; 7 Departments of Ophthalmology, Neurology, and Neurosurgery, Weill Cornell Medicine, New York, NY USA; 8 Department of Ophthalmology, University of Texas Medical Branch, Galveston, TX USA; 9 University of Texas MD Anderson Cancer Center, Houston, TX USA; 10 Texas A&M College of Medicine, Bryan, TX USA; 11 Department of Ophthalmology, The University of Iowa Hospitals and Clinics, Iowa City, IA USA

**Keywords:** COVID-19, Ophthalmic disease, Ophthalmology

## Abstract

**Background:**

Anecdotally, the COVID-19 pandemic has resulted in more severe cases of eye disease, decreased medication compliance/availability, and decreased treatment volume due to the lockdown.

**Aims:**

We aim to quantify and bring together a variety of international perspectives from ophthalmologists of different subspecialties to characterize the global impact of COVID-19 on managing various ophthalmic disease.

**Methods:**

An online survey of 10 questions was conducted among ophthalmologists using a specialized survey program.

**Results:**

Fifty-two ophthalmologists were successfully contacted. Survey respondents include ophthalmologists from USA, Canada, Korea, Mexico, and New Zealand. Based on the results of our survey, 1 year after the pandemic, ophthalmic disease severity has worsened as well as a decrease in examination and medication compliance.

**Conclusions:**

Ophthalmologists across the world have reported a general increase in disease severity and decrease in medication and examination compliance 1 year after the beginning of COVID-19.

During the rise of COVID-19 in March 2020, the American Academy of Ophthalmology (AAO) recommended for ophthalmologists to “cease any treatment other than urgent or emergent eye care immediately” [1]. After it was clear that the pandemic would persist, AAO called for a careful re-opening 1 month later, due to the immense need to see patients to address preventable blindness. Most ophthalmic services worldwide followed similar treatment patterns. Anecdotally, the COVID-19 pandemic has resulted in more severe cases of eye disease, decreased medication compliance/availability, and decreased treatment volume due to the lockdown. In this descriptive study, we aim to quantify and bring together a variety of international perspectives from ophthalmologists of different subspecialties to characterize the global impact of COVID-19 on managing various ophthalmic disease.

## Data collection

Approval was obtained from the Institutional Review Board at the University of Nevada Reno. An online survey of 10 questions was conducted among ophthalmologists using a specialized survey program (Momentive Inc., USA). The survey was open for 68 days from August 26 to October 9, 2021. A total of 52 ophthalmologists from 5 different countries, across 4 continents, responded to the questionnaire. The ophthalmology subspecialties were well represented within this survey with respondents including 30 neuro-ophthalmologists, 7 comprehensive, 3 cornea, 1 glaucoma, 6 retina, 1 uveitis, 3 pediatrics, and 1 ophthalmology resident.

## Results

**Fig. 1:**
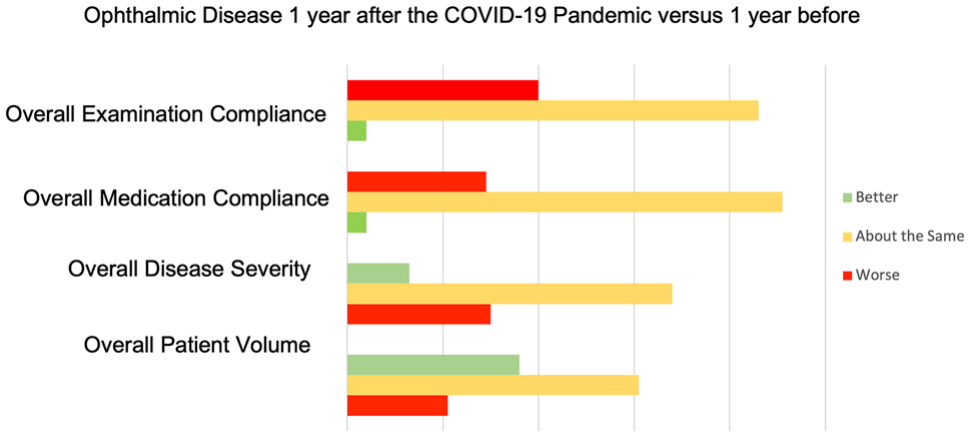
Examining the impact of the COVID-19 pandemic on various commonly seen ophthalmic conditions. The red represents negative responses, such as an increase in patient volume, or a decrease in patient medication compliance

**Fig. 2:**
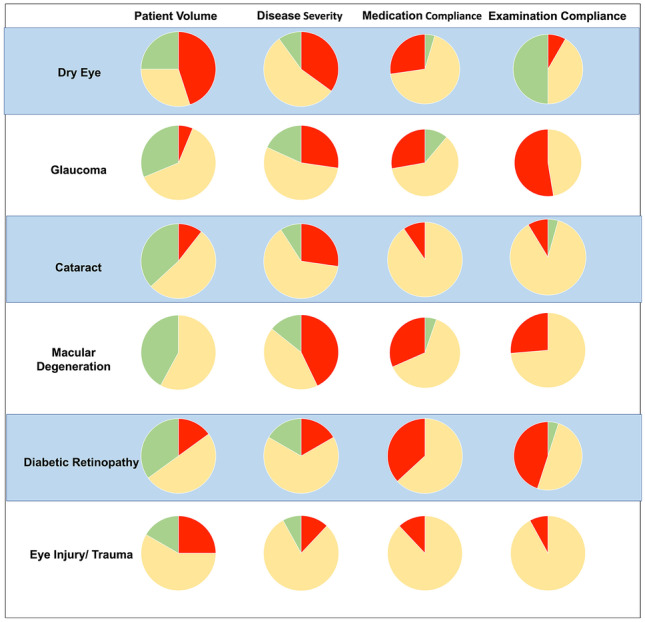
Specific breakdown of survey responses by ophthalmic condition. Note the impact on disease severity and medication compliance differs significantly by condition. Red indicates worsening, yellow indicates about the same, green indicates an improvement

Of 52 ophthalmologists successfully contacted, survey respondents include ophthalmologists from USA, Canada, Korea, Mexico, and New Zealand. The average age of a survey respondent was 51 years old. Most participants worked in universities or public hospitals 56% (*n* = 29), while 44% worked in private practice, either solo or in a group (*n* = 23) (Figs. 1 and 2).

## Discussion

The COVID-19 pandemic has created a significant barrier to eye care in regions that previously had an abundance of ophthalmic services available. Based on the results of our survey, 1 year after the pandemic, ophthalmic disease severity has worsened as well as a decrease in examination and medication compliance. The disease severity for macular degeneration significantly increased likely due to missed anti-VEGF injection appointments, while eye injury and trauma remained essentially unchanged.

The decrease in examination compliance has shown the importance of improving the quality of remote eye care and teleophthalmology capabilities. At-home optical coherence tomography (OCT) is an emerging technology that has shown promise in improving remote eye care [2]. Extended reality-based technology for ophthalmic assessments, such as visual fields, has also been deployed and has shown encouraging results during the pandemic [3]. While this study provides an international perspective from ophthalmologists across different specialties and various practice patterns, a limitation to this descriptive study is the sample size and minimal comparative statistics. Further studies with increased sample sizes are required to examine the impacts of the COVID-19 on ophthalmic practices and disease severity. Further research is also required on the development of at-home visual assessment technologies for future pandemics [4–7].

## Conclusion

Ophthalmologists across the world have reported a general increase in disease severity and decrease in medication and examination compliance 1 year after the beginning of COVID-19.

## Data Availability

Data is available upon reasonable request.
